# Supplementary material for: Human mobility in a Bronze Age Vatya ‘urnfield’ and the life history of a high-status woman
Source: PLoS One. 2021 Jul 28;16(7):e0254360. doi: 10.1371/journal.pone.0254360 (PMC8318297; doi:10.1371/journal.pone.0254360)
Supplement: S1 File — (DOCX) [file pone.0254360.s001.docx]

**Radiocarbon dates**

Radiocarbon dates from the Szigetszentmiklós-Ürgehegy cemetery have been produced for 11 burials: nine cremations and two inhumations. It is well-known that dating of cremation burials can be challenging, since cremated bones can provide slightly older or younger dates (e.g. due to the ‘old wood effect’ of the pyre, or to other diagenetic processes) than collagen samples of the same period (Snoeck, Brock and Schulting, 2014; Dani *et al.*, 2019; Major *et al.*, 2019). In our case cremated remains provided somewhat later dates for “phase 1” and “phase 2” between 1870 and 1450 BC, e.g. burial 241 were dated between 1740 and 1500 BC, while grave 64 of the cemetery excavated at Szőreg (of the Maros culture) containing a similar bone needle and contemporaneous with our “phase 1”, was recently dated between 2030 and 1960 BC (Bóna, 1975, fol. 119.14; O’Shea *et al.*, 2019). On the contrary, inhumations do not show particular issues and can therefore be considered reliable.

| **Grave N.** | **AMS ^14^C lab. code** | **Rite** | **Sex** | **Age** | **2σ cal.** | **Phase (chronotypology)** |
| --- | --- | --- | --- | --- | --- | --- |
| 190 | DeA-26794 | I | U | 30-40 | 1550-1290 BC | NA |
| 224 | DeA-27289 | C | F | 20-40 | 1630-1430 BC | 3 |
| 241a | DeA-27290 | C | F | 25-35 | 1740-1500 BC | 1 |
| 350 | DeA-27291 | C | U | c. 5 | 1500-1260 BC | 2 |
| 433 | DeA-27292 | C | U | 20-40 | 1630-1420 BC | 3 |
| 440 | DeA-27293 | C | M | 20-30 | 1740-1450 BC | 2 |
| 449 | DeA-27294 | C | U | c. 5 | 1750-1500 BC | 3 |
| 459 | DeA-27295 | C | F? | 18-25 | 1390-1050 BC | 1 |
| 466 | DeA-27296 | C | F | 15-20 | 1750-1500 BC | 1 |
| 480 | DeA-27297 | C | U | c. 6 | 1870-1510 BC | 2 |
| 489 | DeA-27411 | I | U | 30-40 | 1690-1500 BC | NA |

Table 1. List of radiocarbon dates.


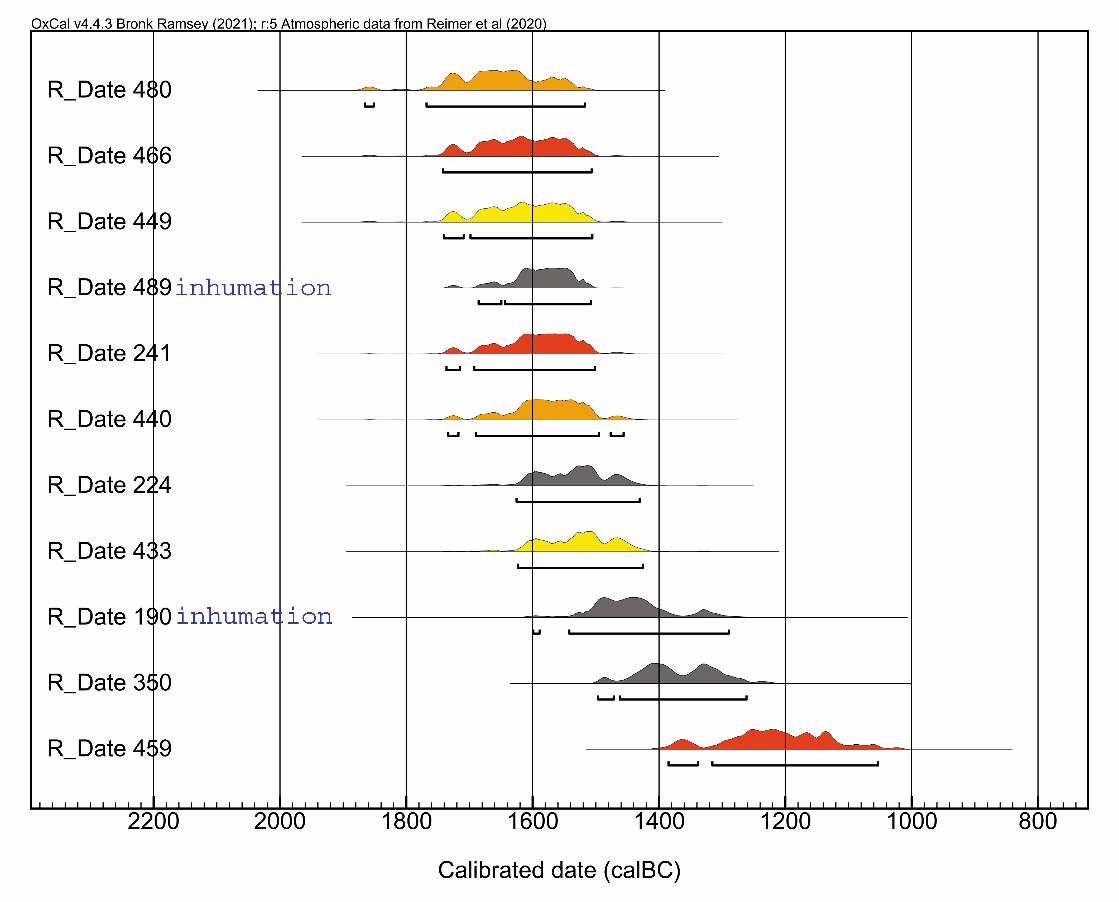


Fig 1. Sequence of radiocarbon dates. Colours represent the chronological phases identified on the basis of typology (red=“phase 1”; orange=”phase 2”; yellow=”phase 3”; grey=”not datable”).
