# Supplementary figures and images for: Human mobility in a Bronze Age Vatya ‘urnfield’ and the life history of a high-status woman
Source: PLoS One. 2021 Jul 28;16(7):e0254360. doi: 10.1371/journal.pone.0254360 (PMC8318297; doi:10.1371/journal.pone.0254360)

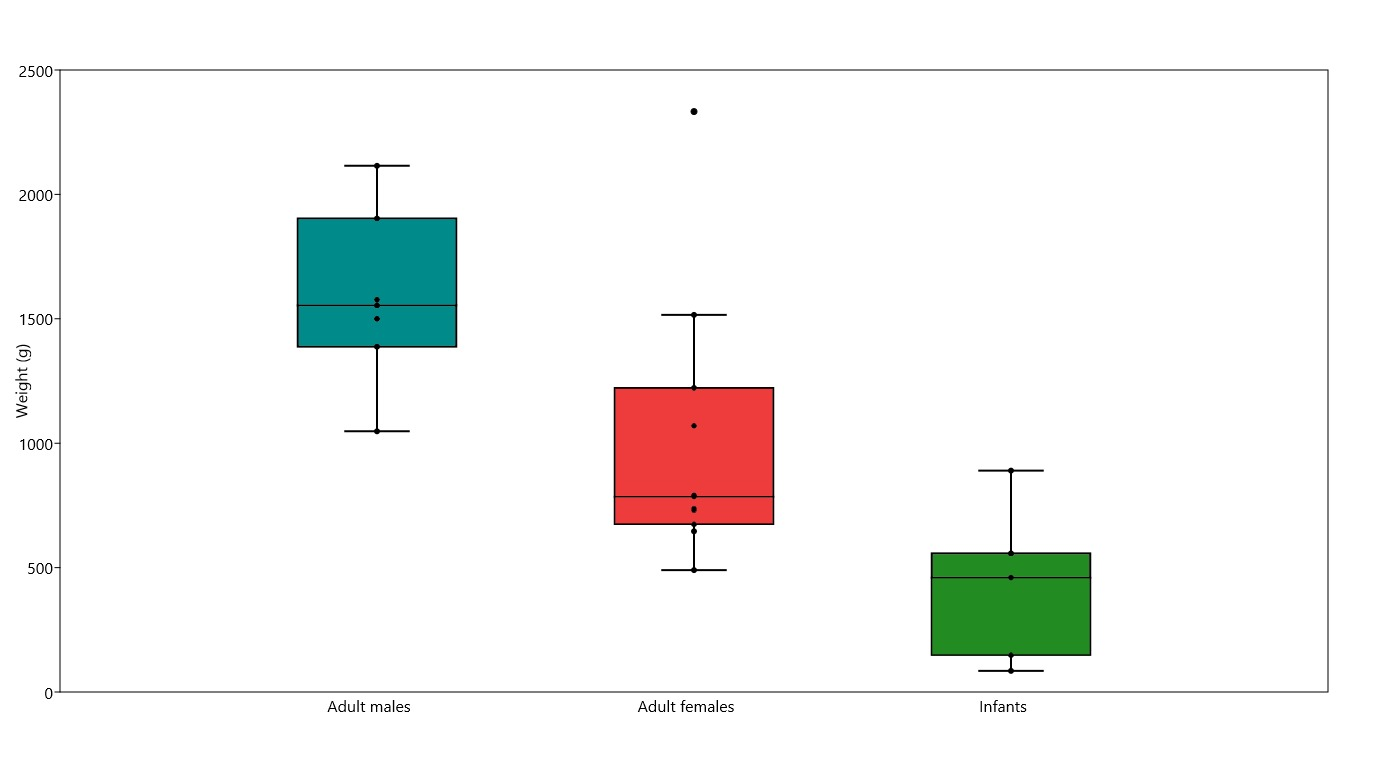

Supplement: S1 Fig — (TIF) [file pone.0254360.s002.tif]

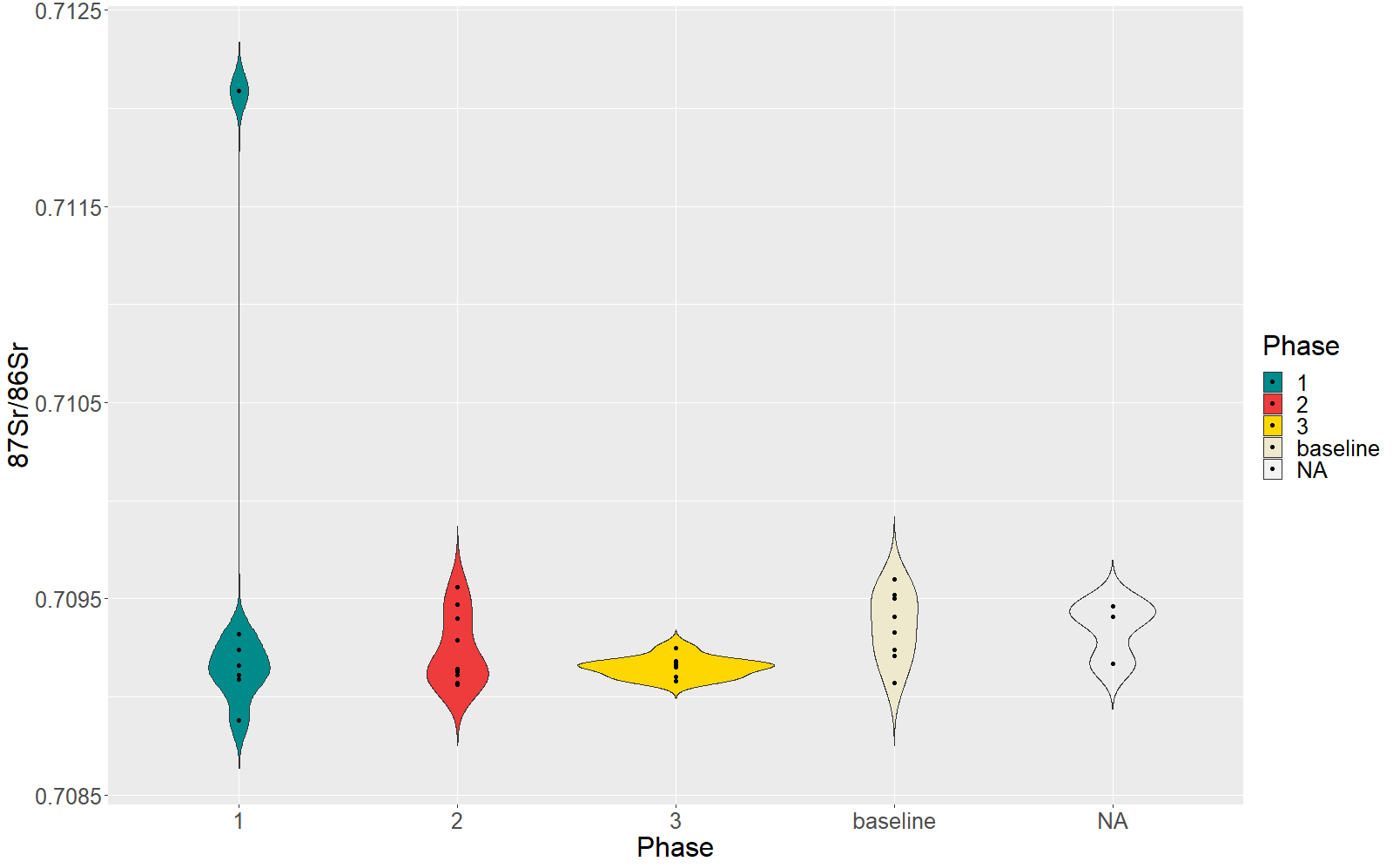

Supplement: S2 Fig — Distribution of the 87Sr/86Sr values of burials in the various phases compared with local baselines at Szigetszentmiklós. (TIFF) [file pone.0254360.s003.tiff]

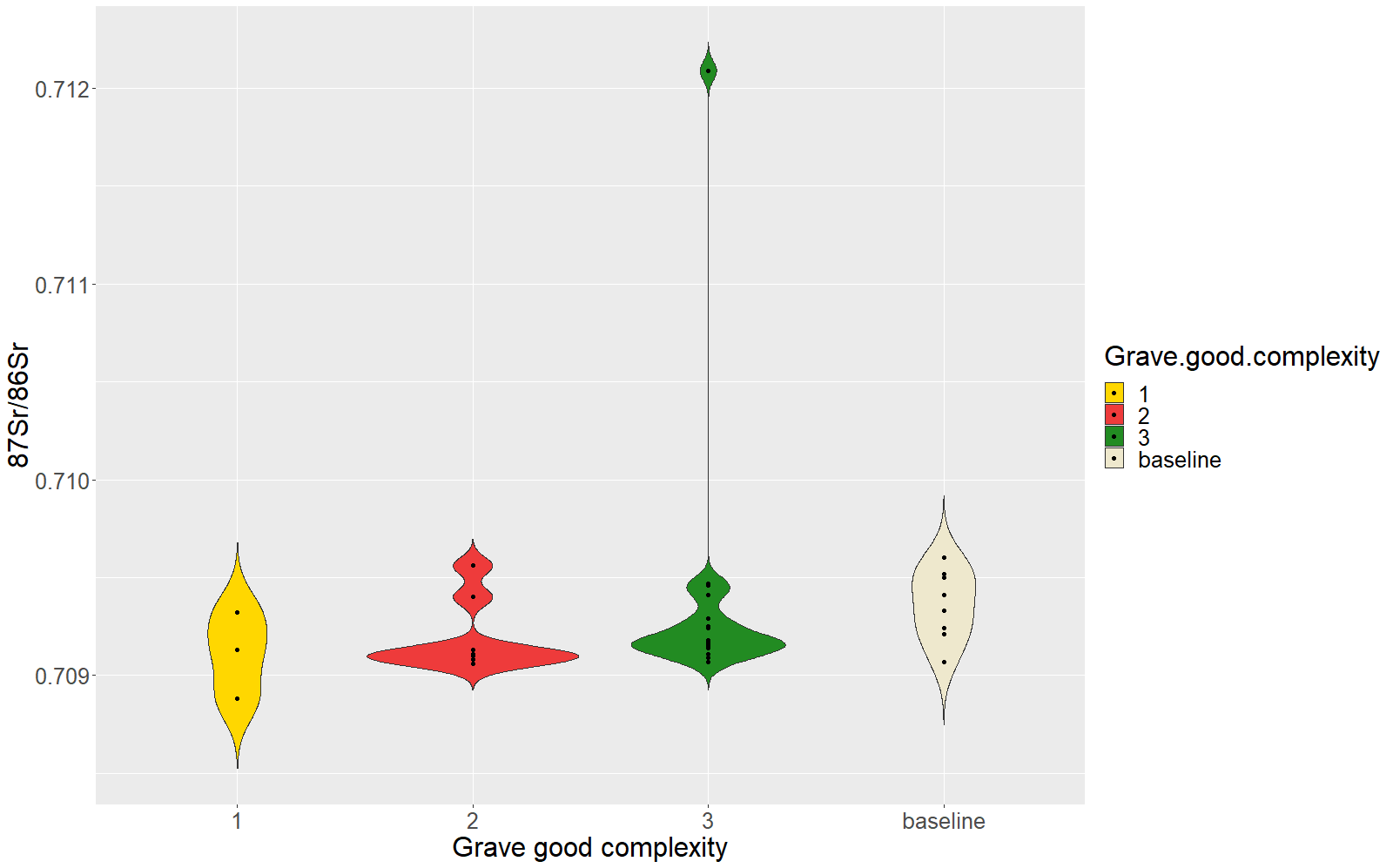

Supplement: S3 Fig — Distribution of the 87Sr/86Sr values of burials with different grave good complexity compared with local baselines at Szigetszentmiklós. (TIFF) [file pone.0254360.s004.tiff]

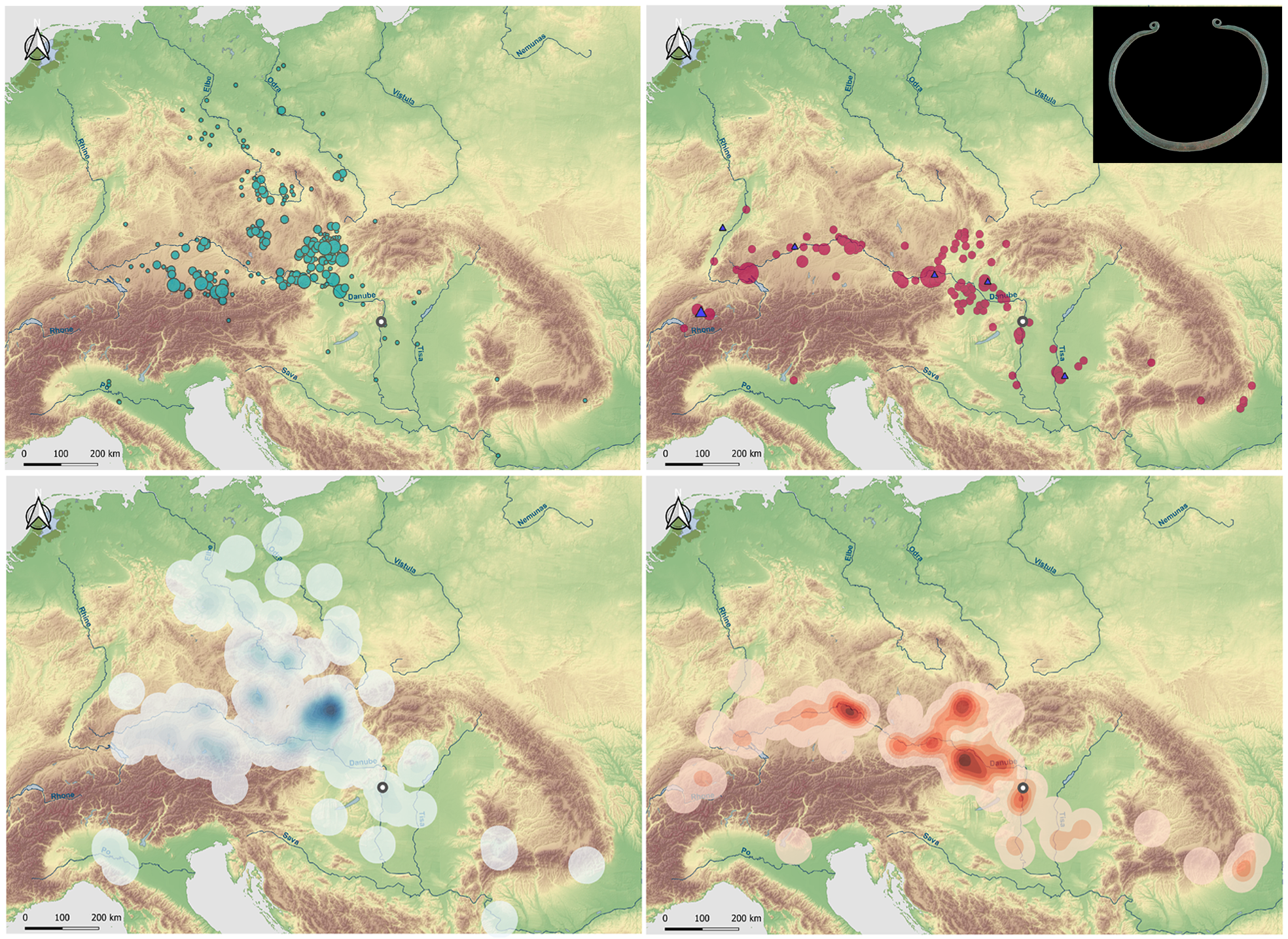

Supplement: S4 Fig — Top left: Geographical distribution of the Ösenringe hoards; bottom left: Density distribution of the Ösenringe in hoards (data gathered from [26, 134, 136, 138, 156]). Top right: Geographical distribution of the Ösenringe in burials; bottom right: Density distribution of the Ösenringe in burials (data gathered from [21, 26, 144, 148]). The maps are constructed using “Natural Earth. Free vector and raster map data @ naturalearthdata.com” available at https://www.naturalearthdata.com/downloads/10m-raster-data/ (TIF) [file pone.0254360.s005.tif]

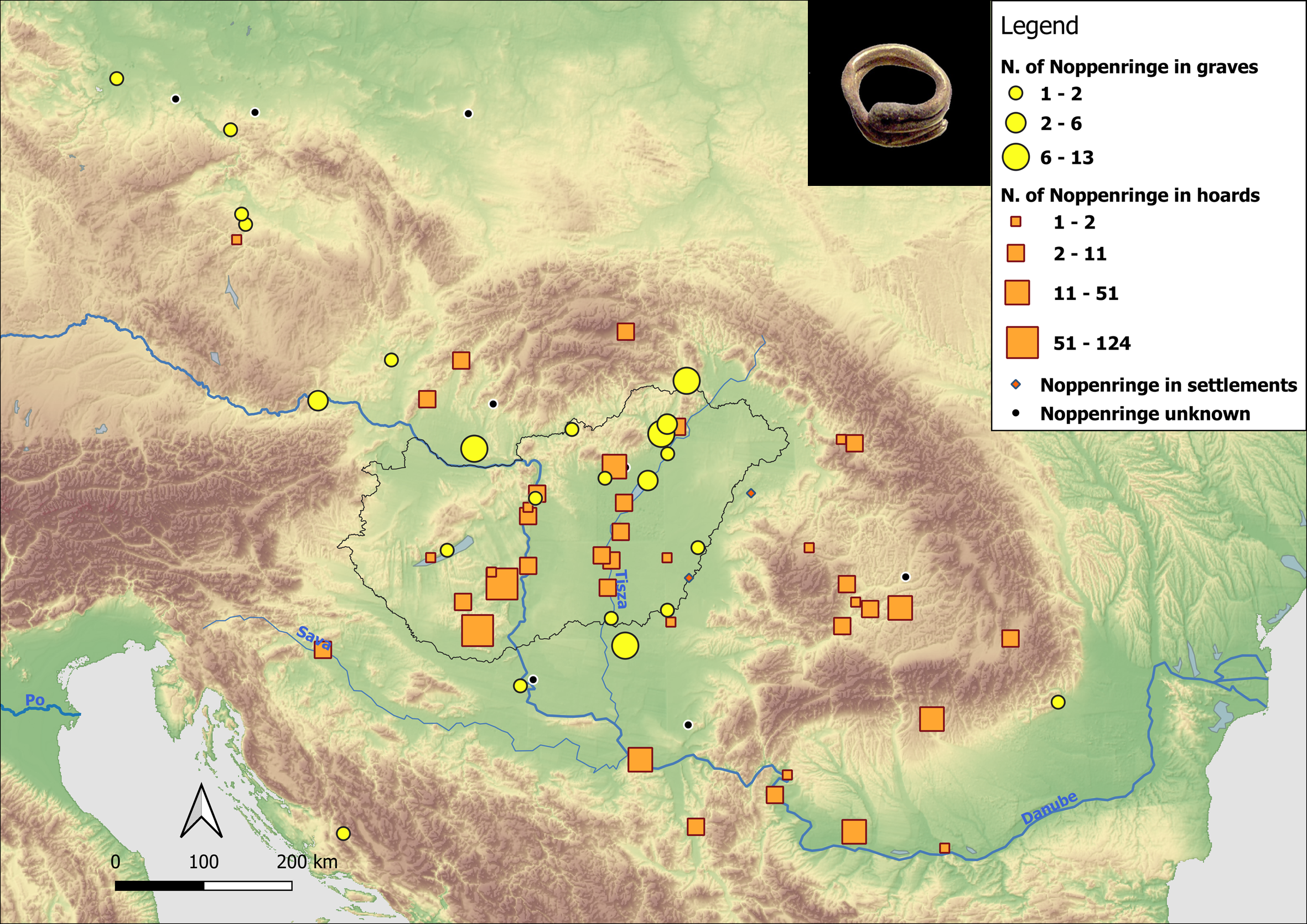

Supplement: S5 Fig — The map is constructed using “Natural Earth. Free vector and raster map data @ naturalearthdata.com” available at https://www.naturalearthdata.com/downloads/10m-raster-data/,. (TIF) [file pone.0254360.s006.tif]
