# Supplementary material for: Human mobility in a Bronze Age Vatya ‘urnfield’ and the life history of a high-status woman
Source: PLoS One. 2021 Jul 28;16(7):e0254360. doi: 10.1371/journal.pone.0254360 (PMC8318297; doi:10.1371/journal.pone.0254360)
Supplement: Attachment — Submitted filename: Response to Reviewers.docx [file pone.0254360.s008.docx]

Dear Editor and Reviewers,

we are very grateful to you for the attention given to our manuscript. We changed the figures, tabs, caption and text according to your suggestions.

Also on behalf of my co-authors, thanks again for your efforts,

Yours,

Claudio Cavazzuti

EDITOR

Names of site and rivers are hardly readable on the map of figure 1 and a contextualization map should be added as embedded picture.

- We enlarged river labels.

Please add some scale reference for figure 2, 4 and 5.

- Done.

Does Figure 5 show the bones of both foetuses (see caption)?

- We changed the caption to “Bones attributable to both foetuses (n. 241b and 241c).”

Could it be combined with Figure 4?

- Done. We changed all the following figures and reference to figures in the text and captions.

In figure 7, in addition to add axis lines, please have the number given in a consistent way (0.7090 instead of 0.709 along the y axis of the bottom graph).

- Done.

Figures 8 and 9 present a y axis with numbers including only three decimals, while 4 (even 5 in Figure 10) decimals are given elsewhere.

- Done. We made them homogenous.

I suggest transferring Figure 11 and 12 in the supplementary data (please note that the name rivers are again difficult to read) and to add an illustration (picture, drawing) of bronze neck-ring and gold ring as embedded picture (e.g. top right of the map) to have a better visualization of the conveyed information.

- Done. Unfortunately, we do not have the possibility of changing the font size of river names, but as it is a digital version, one can zoom in, in case.

REV2

The abstract mentions 29 individuals, while the MM section refers to 30.

- Thanks. We changed the MM section as follows: “We collected a total of 41 samples from different human tissues included in 29 burials (3 inhumations and 26 urn cremations; burial n. 241 includes three individuals, two of them analysed), as well as from 3 bone pins/needles from burials n. 215 and 241a (Table 1).”

Supplementary data 14C: could you add the sample ID of the 14C laboratory?

- Yes, we did it.

Supplementary data 14C: chronotipology ◊ chronotypology

- Changed

Supplementary data 14C: age (in years). Ad = ?
- We changed to “20-40”

Few suggestions
Fig 7: density plot: could you add the X-axis?

- Done

Fig 7: graph: could you put 87 and 86 in superscript and replace the commas (,) with periods (.)

- Done

Fig 8-9-10: ditto in superscript

- Done

Suppl.3-4: ditto in superscript

- Done
